# Psychological traits associated with anabolic androgenic steroid use and dependence: an exploratory cross-sectional study among female athletes

**DOI:** 10.1186/s12905-025-03711-5

**Published:** 2025-05-05

**Authors:** Morgan Scarth, Ingrid Amalia Havnes, Marie Lindvik Jørstad, Astrid Bjørnebekk

**Affiliations:** 1 Anabolic Androgenic Steroid Research Group, Section for Clinical Addiction Research, Division of Mental Health and Addiction, Oslo University Hospital, Postbox 4959, Nydalen, Oslo, 0424 Norway; 2 Division of Mental Health and Addiction, Oslo University Hospital, Oslo, Norway; 3 Institute of Clinical Medicine, University of Oslo, Oslo, Norway; 4 Department of Substance Use and Addiction Treatment, Oslo University Hospital, Oslo, Norway

**Keywords:** Anabolic androgenic steroids, Testosterone, Attention deficits, Aggression, Dependence

## Abstract

**Background:**

Anabolic–androgenic steroids (AAS) are commonly taken to increase muscle size and enhance performance. However, AAS can lead to many adverse effects, including challenges with mental health and behavior. This study aims to identify behavioral and psychological correlates of AAS use, and explore associations with dependence symptoms among female weightlifters.

**Methods:**

A sample of *n* = 32 female weightlifters, including 16 with reported AAS use completed questionnaires including the Achenbach System of Empirically Based Assessment (ASEBA) and Buss-Perry Aggression Questionnaire (BPAQ). AAS dependence was evaluated using the Structure Clinical Interview for DSM-IV. Group comparisons were made using Welch’s t-tests between control and AAS groups, and AAS dependent and non-dependent groups. Exploratory correlation analyses were computed between symptoms of dependence and behavioral and psychiatric scales.

**Results:**

Females who had used AAS had higher levels of both externalizing and internalizing psychopathology, including antisocial and attention problems, as well as aggressive traits. The most prevalent dependence symptoms were time spent on activities surrounding AAS use (*n* = 7, 50%), and using more or for longer than planned (*n* = 6, 42.9%). Dependence symptoms were associated with several ASEBA scales: tolerance was correlated with aggressive behavior (*ρ* = 0.79, *p* < 0.001), withdrawal was correlated with attention problems (*ρ* = 0.78, *p* < 0.001), and being unable to quit use was associated with anxious/depressive (*ρ* = 0.80, *p* < 0.001) and internalizing problems (*ρ* = 0.79, *p* < 0.001).

**Conclusions:**

Females who currently or previously used AAS demonstrated significantly more difficulties with maladaptive functioning and aggressive traits compared to those who have never used. Attention problems and aggressive behavior were also associated with symptoms of AAS dependence, though longitudinal studies are required to determine the direction of this relationship.

**Supplementary Information:**

The online version contains supplementary material available at 10.1186/s12905-025-03711-5.

## Background

Anabolic androgenic steroids (AAS) are synthetic derivatives of testosterone that are most commonly used by athletes and bodybuilders to enhance physical performance and increase muscle mass. The global lifetime prevalence of AAS use is estimated to be 1.6% for females and 6.4% for males, although prevalence is elevated within certain subpopulations, including bodybuilders, people in prison, and people with substance use disorders (SUD) [1–3]. A recent meta-analysis reported that 1.4% of women in the general population reported using AAS, while 16.8% of female bodybuilders reported AAS use [4]. While AAS use is more common among males, use may increase among females as a result of shifting body ideals towards a more lean and muscular physique [5–7]. However, females using AAS face additional stigma relative to their male counterparts, which may contribute to underreporting and distorted prevalence estimates [8–10]. In addition, women using AAS may be susceptible to low-quality information regarding their health and risks, as many report relying on advice from unqualified partners, coaches, or online sources [11]. Risks associated with female AAS use include sex-specific adverse effects such as changes to menstruation, clitoral enlargement, voice deepening and reduced breast volume, in addition to physical and psychological side-effects which have been well-established in males including cardiovascular abnormalities, acne, anxiety, reduced fertility and aggressive behavior [9, 12–18]. Females may seek health care for AAS related symptoms earlier than males, suggesting potentially more noticeable or concerning side effects [8].

As the majority of research on AAS use has focused on males, there is a lack of knowledge around risk factors for, and consequences of AAS use in females, particularly regarding the effects of AAS on the brain. Among males, studies have identified associations between AAS use and a number of cognitive, neurobiological, and psychiatric challenges including decreased executive function, cortical thinning, accelerated brain aging, and increased internalizing and externalizing psychopathology [19–24]. Similarly, there is likely an increased prevalence of psychiatric disorders among females who use AAS [25], in addition to potential social cognitive challenges [26]. Females may also be more vulnerable than males to certain psychiatric side effects. For example, animal studies indicate that differences in neural circuits associated with anxiety-like behaviors in females are more sensitive to the effects of exogenous androgens than in males [27].

Approximately 30% of men who use AAS develop dependence, characterized by withdrawal symptoms and continued use despite side effects, as a result of prolonged use and increased dosage [28–30]. AAS dependence among men is associated with experiencing more adverse effects, psychopathology, attention problems, and aggressive behavior relative to men without dependence [15, 19, 30–32]. However, few studies have investigated AAS dependence and its correlates in a female sample. In animal models, AAS appear to be rewarding in both males and females, as female hamsters will self-administer testosterone to a similar extent as males [33, 34]. This finding suggests that females have a similar potential to develop dependence despite differences in societal norms, however this has not been confirmed in human studies [33]. One study among 12 females using AAS indicated that 58.3% of the sample met criteria for performance-enhancing substance dependence, which was significantly higher than the male sample (23.4%) [18]. In the same study, a higher proportion of females who used AAS reported attention-deficit/hyperactivity disorder (ADHD) diagnoses compared to non-using females. While it is not well established whether attention problems represent a risk factor for AAS dependence in women, ADHD is frequently comorbid with dependence of other drugs of abuse in women [35].

Internalizing and externalizing pathologies, including aggression, may also be elevated among women who use AAS compared to non-using women [25, 36]. While relatively few studies have measured aggression among females using AAS, some women have reported aggressive behaviors, labile mood, and irritability as a result of their use [37]. Among adolescents, female AAS use was found to be associated with increased fighting [38]. However, one study found that there were no differences in aggression between male and females who used AAS [39], while another found that females demonstrated lower levels of aggression relative to males [40]. Animal studies suggest putative sex differences in the effects of AAS on neural circuitry underlying aggressive behaviors, though these associations are complex and vary with the type of AAS administered as well as environmental factors [41]. Thus, the effects of AAS on aggressive behavior in women are not well understood. Additionally, while higher levels of aggression have been identified among males with AAS dependence compared to both non-using and non-dependent men [15], there is a knowledge gap regarding the relationship between AAS dependence and aggression among women.

The purpose of this study is to investigate the relationship between AAS use and psychological factors among women, and to explore potential associations of these factors with dependence symptoms. Specifically, we aim to evaluate the degree to which behavioral, emotional and social problems including aggressive traits and attention problems are associated with female AAS use and dependence.

## Methods

### Study design

This study uses data from a larger mixed-methods study investigating brain and psychological aspects associated with use of AAS at Oslo University Hospital. Participants provided data via questionnaires, interviews, and neuroimaging exams.

### Participant characteristics

The final sample comprised 32 participants (*n* = 16 AAS, *n* = 16 WLC), and *n* = 1 WLC was removed due to a positive test for a WADA banned performance enhancing substance not categorized as AAS. Within the AAS group, six females were currently using, and ten reported previous use. Use status was confirmed via urine samples, as has been previously described [25]. The mean age of participants in the WLC group was 28.44 years (SD = 4.50), and in the AAS group, 31.00 years (SD = 7.68). Participants in the WLC group had, on average, 15.97 years of education (SD = 2.09), while those in the AAS group had 14.41 years (SD = 2.06). Bodybuilding was the most common form of training in both groups, comprising 43.8% of the WLC group and 31.2% of the AAS group. Demographic variables and information about AAS and other performance enhancing drug use are given in Table 1.

**Table 1:** Sample characteristics

	**WLC**	**AAS**		
	*n=*16	*n*=16	*t*	*p*
Age (mean (SD))	28.44 (4.50)	31.00 (7.68)	−1.15	0.259
Education (years)	15.97 (2.09)	14.41 (2.06)	2.13	0.042
Alcohol units/week (mean (SD))	0.66 (0.91)	1.73 (2.73)	−1.43	0.146
Height (cm)	167.22 (7.80)	166.41 (6.51)	0.32	0.751
Weight (kg)	65.48 (9.76)	65.91 (9.81)	−0.12	0.903
Positive test (n (%)	0 (0.0)	5 (38)^a^		0.01^†^
T/E ratio (mean (SD))	1.09 (0.67)	1.25 (1.11)		0.627
*Training type (n(%))*				0.659^†^
Bodybuilding	7 (43.8)	5 (31.2)		
Powerlifting	2 (12.5)	1 (6.2)		
Combat sports	1 (6.2)	2 (12.5)		
Recreational sports	5 (31.2)	8 (50.0)		
Other	1 (6.2)	0 (0.0)		
*Training intensity (mean (SD))*				
Minutes strength/week	356.17 (214.69)	357.14 (299.90)	−0.01	0.992
Minutes endurance/week	116.00 (115.56)	170.00 (140.18)	−1.07	0.283
Squat record	98.25 (20.69)	112.73 (33.64)	−1.23	0.223
Squats current max	96.64 (17.56)	81.25 (35.33)	1.13	0.226
Bench record	72.65 (9.61)	77.71 (24.85)	−0.65	0.552
Bench current max	62.00 (23.14)	63.81 (29.05)	−0.15	0.878
Deadlift record	117.14 (30.30)	106.36 (24.09)	0.99	0.345
Deadlift current max	108.36 (40.25)	85.62 (34.38)	1.40	0.195
**Characteristics of AAS use**				
Debut age (years) (median (IQR))		22.00 [20.75, 26.25]		
Total years AAS use		1.50 [1.00, 4.00]		
Number of cycles^b^		3.00 [1.50, 4.25]		
Weekly dose (mg)^b^		240.00 [190.00, 420.00]		
Cycle duration (weeks)^b^		9.00 [7.00, 17.00]		
**Current/previous AAS (n (%))**				
Previous consumer		9 (56.2)		
*Months since quitting (median [IQR])*^*a*^		16 [7.50, 41.00]		
Current consumer		7 (43.8)		
*Number of compounds*^*c*^		7 (4.75)		
**Compounds used (n (%))**^**c**^				
Anavar (Oxandrolone)		12 (80.0)		
Winstrol (Stanozolol)		10 (66.7)		
Primobolan (Metenolone)		10 (66.7)		
Nandrolone		7 (46.7)		
Clenbuterol*		11 (73.3)		
Growth hormone*		8 (53.3)		

^a^3 missing

^b^5 missing

^c^1 missing

^†^Fisher’s exact test

^*^non-AAS performance enhancing drugs

### Measures

#### Adaptive and maladaptive functioning

Adaptive and maladaptive functioning in the previous six months were evaluated with the Adult Self-Report (ASR) form of the Achenbach System of Empirically Based Assessment (ASEBA) [42]. The ASR consists of 126 items rated on a 3-point scale (“not true”, “somewhat to sometimes true”, and “very true or often true”). The items were summed for each of the following scales: anxious/depressed, withdrawn, somatic complaints, thought problems, attention problems, aggressive behavior, rule-breaking behavior, and intrusive behavior. Six DSM-oriented scales were computed: depressive problems, anxiety problems, somatic problems, avoidant personality problems, attention deficit/hyperactivity problems (inattention and hyperactivity/impulsivity subscales), and antisocial personality problems. Internalizing problems are indicated by the sum of the anxious/depressed, withdrawn and somatic complaints scales, and externalizing problems are indicated by the sum of the aggressive, rule-breaking and intrusive behavior scales. Higher raw scores indicate more problematic behaviors for maladaptive scales. Normalized T-scores, weighted for sex and age, were computed, where a clinically significant threshold is indicated by T-scores ≥ 70, while scores ranging from 65 to 69 are considered “borderline”. In addition, the ASR contains 38 items summed into five scales measuring adaptive functioning (friends, spouse/partner, family, job, and education). The Cronbach's alpha for the scales ranged from 0.56 to 0.95, indicating poor to excellent reliability (Table 2).

**Table 2:** Group mean and standard deviations of Buss-Perry Aggression Questionnaire and Achenbach System of Empirically Based Assessment

	**Mean (SD)**	**Mean (SD)**	**α**	**t**	***d***	***p***	***p***_***FDR***_
	**WLC, *n* = 16**	**AAS, *n* = 16**					
**Buss-Perry**^**a**^							
Physical aggression	15.77 (4.94)	23.57 (8.78)	0.70	− 2.87	1.08	**0.009**	**0.018**
Verbal aggression	17.08 (6.08)	18.79 (5.21)	0.65	− 0.78	0.30	0.442	0.500
Anger	14.23 (4.71)	25.00 (8.04)	0.82	− 4.28	1.62	**0.000**	**0.001**
Hostility	15.08 (5.96)	24.29 (9.79)	0.83	− 2.97	1.13	**0.007**	**0.015**
Total aggression	62.15 (17.84)	91.64 (18.19)	0.87	− 4.25	1.64	**0.000**	**0.001**
**ASEBA (T-scores)**							
Anxious/depressive	53.31 (5.16)	65.25 (12.48)	0.93	− 3.54	1.25	**0.002**	**0.005**
Withdrawn	53.06 (4.65)	58.62 (9.11)	0.76	− 2.18	0.77	**0.040**	0.066
Somatic complaints	55.06 (5.84)	60.88 (10.95)	0.84	− 1.87	0.66	0.074	0.107
Thought problems	52.81 (4.87)	57.50 (7.03)	0.56	− 2.19	0.77	**0.037**	0.065
Attention problems	54.00 (3.69)	64.62 (6.74)	0.83	− 5.53	1.96	**0.000**	**0.000**
Aggressive behavior	50.75 (1.29)	60.50 (8.16)	0.87	− 4.72	1.67	**0.000**	**0.001**
Rule breaking	50.81 (1.72)	60.38 (8.80)	0.83	− 4.27	1.51	**0.001**	**0.002**
Intrusive	51.31 (2.09)	54.31 (5.20)	0.59	− 2.14	0.76	**0.045**	0.069
Internalizing	47.94 (11.09)	62.31 (13.73)	0.95	− 3.26	1.15	**0.003**	**0.006**
Externalizing	42.06 (7.23)	59.81 (10.20)	0.91	− 5.68	2.01	**0.000**	**0.000**
Depressive	52.81 (3.95)	64.56 (11.43)	0.87	− 3.89	1.37	**0.001**	**0.003**
Anxious	51.19 (2.71)	57.62 (9.12)	0.82	− 2.71	0.96	**0.015**	**0.027**
Somatic problems	53.06 (4.70)	57.31 (10.42)	0.84	− 1.49	0.53	0.152	0.197
Avoidant	54.50 (5.68)	57.31 (7.29)	0.60	− 1.22	0.43	0.234	0.289
ADHD	54.38 (6.76)	68.44 (10.12)	0.88	− 4.62	1.63	**0.000**	**0.001**
Antisocial	51.12 (1.89)	62.62 (8.48)	0.83	− 5.29	1.87	**0.000**	**0.001**
Friends	52.62 (5.50)	50.19 (7.87)	0.43	1.02	0.36	0.319	0.377
Spouse/partner^b^	52.60 (5.02)	51.00 (7.01)	0.73	0.54	0.27	0.596	0.646
Family	45.19 (10.29)	45.19 (10.89)	0.74	0.00	0.00	1.00	1.00
Job^c^	50.69 (8.94)	45.55 (6.22)	0.65	1.76	0.65	0.090	0.123
Education^d^	47.83 (11.36)	46.33 (7.17)	0.58	0.27	0.16	0.791	0.823

α = Cronbach’s α, t = Welch’s t-statistic, p adjusted = false discovery rate adjusted *p*-value

^a^missing n = 3 WLC, n = 2 AAS

^b^no spouse/partner n = 6 WLC, 8 AAS

^c^not currently working n = 5 AAS

^d^not currently a student n = 10 AAS, 10 WLC

#### Aggression

The Buss-Perry Aggression Questionnaire (BPAQ) was administered to evaluate aggressive traits. The self-report questionnaire contains 29 items producing a total aggression score which is the sum of four subscales: physical aggression, verbal aggression, anger and hostility [43]. The Cronbach's alpha for the scales ranged from 0.65 to 0.87, indicating acceptable to good reliability (Table 2).

#### AAS dependence

AAS dependence was measured using the Structured Clinical Interview for DSM-IV Axis II Disorders (SCID-II) for substance dependence, with adaptations for AAS by experts in the field, which has been found to have sufficient reliability and validity [29]. Lifetime AAS dependence was evaluated based on seven symptoms, which are described in Table 3. Trained study personnel conducted interviews, and rated each symptom on a scale from 1–3 (absent, subthreshold, present). Participants were considered *dependent* if they presented with three or more symptoms. In subsequent analyses, the total number of AAS dependence symptoms were summed to approximate severity of dependence.

**Table 3:** Description of AAS dependence symptoms and prevalence of each symptom among women who used AAS (*n* = 14)

**Symptom**	**Level**	**N (%)**
Tolerance		
*A need for markedly increasing amounts of the substance to achieve desired effect, or markedly diminished effect with continued use of the same amount of the substance*	*Absent*	6 (42.9)
	*Subthreshold*	5 (35.7)
	*Present*	3 (21.4)
Withdrawal		
*As manifested by either of the following: the characteristic withdrawal syndrome: depressed mood, fatigue, insomnia. AAS are used to relieve or avoid withdrawal symptoms*	*Absent*	10 (71.4)
	*Subthreshold*	2 (14.3)
	*Present*	2 (14.3)
Use longer than planned		
*The substance is often taken in larger amounts or over a longer period than was intended*	*Absent*	6 (42.9)
	*Subthreshold*	2 (14.3)
	*Present*	6 (42.9)
Unable to stop		
*Persistent desire or unsuccessful efforts to cut down or control substance use*	*Absent*	8 (57.1)
	*Subthreshold*	3 (21.4)
	*Present*	3 (21.4)
Time spent		
*A great deal of time is spent in activities necessary to obtain the substance, use the substance, or recover from its effects*	*Absent*	3 (21.4)
	*Subthreshold*	4 (28.6)
	*Present*	7 (50.0)
Interferes with work/life		
*Important social, occupational, or recreational activities are given up or reduced because of substance use*	*Absent*	7 (50.0)
	*Subthreshold*	2 (14.3)
	*Present*	5 (35.7)
Physical/mental problems		
*The substance use is continued despite knowledge of having a persistent or recurrent physical or psychological problem that is likely to have been caused or exacerbated by the substance*	*Absent*	3 (21.4)
	*Subthreshold*	6 (42.9)
	*Present*	5 (35.7)

### Procedures

This study uses data from a larger mixed-methods study investigating brain and psychological aspects associated with use of AAS at Oslo University Hospital. Participants provided data via questionnaires, interviews, and neuroimaging exams. Participants were recruited through social media and online forums, in addition to informational flyers, posters and snowball sampling. The data collected for this study was part of a larger study including both qualitative and quantitative data, which took place beginning in 2014, and continued until 2018 when an additional 24 participants were added, including the weightlifting control group, to expand the interviews and collect additional data [44]. Participants were included if they were over 18 years of age and participated in heavy resistance training. AAS use was defined as reported current or previous use, and having completed at least one cycle of AAS. Participants who reported no AAS use were categorized as weight-lifting controls (WLC). The sample and data collection process has been previously described in greater detail [9].

#### Statistical analysis

Group differences were evaluated for all measured scales first between the AAS and WLC groups. After assessing normality using Shapiro-Wilks test, group comparisons were evaluated using Welch’s t-test, and Cohen’s d was computed to evaluate effect size, commonly interpreted as small (d = 0.2), medium (d = 0.5), and large (d = 0.8) [45]. Exploratory analyses to investigate group differences in categorized ASEBA scores were conducted using Fisher’s exact tests to compare the number of participants with scores in the normal, borderline, and clinical ranges on ASEBA scales between AAS and WLC groups to account for low expected observations (< 5). Results of statistical tests were considered significant at *p* < 0.05, and p-values were subsequently adjusted for multiple hypotheses (*n* = 26) using Benjamini–Hochberg procedure to control for the false discovery rate (FDR) [46].

Similar exploratory analyses were conducted to compare subgroups of participants who met or did not meet diagnostic criteria for AAS dependence, and for groups reporting current or previous AAS use, to investigate the potential role of quitting use as compared to recent administration of AAS. For each dependence symptom, the proportion of participants endorsing each level (absent, subthreshold, present) was calculated among those who had used AAS with available SCID data (*n* = 14). To investigate the relationship between duration of use and each of the AAS dependence symptoms, Spearman’s rank correlation coefficients were computed. Spearman’s rank correlation coefficient (ρ) was also calculated to investigate associations between SCID items and ASEBA scales which significantly differed between the AAS and WLC groups.

#### Ethics

All participants received a written description of the study prior to enrolling and written formal consent was obtained from all participants. All research was carried out in accordance with the Declaration of Helsinki, and ethical approval was obtained from the Regional Committee for Medical and Health Research Ethics South East Norway (approval #2013/601). The participants were compensated with 1000 NOK (∼100 Euro) for their participation in the study.

## Results

### Buss-Perry and ASEBA group comparisons

The AAS group indicated more aggressive traits relative to WLC on several BPAQ scales, with the largest effects on the anger subscale (*t*(30) = − 4.28, *d* = 1.62, *p*_*FDR*_ = 0.00) and total aggression (mean (SD) AAS = 91.64 (18.19), WLC = 62.15 (17.84), (*t*(30) = − 4.25, *d* = 1.64, *p*_*FDR*_ = 0.00). The AAS group also demonstrated higher T-scores on several internalizing and externalizing scales of the ASEBA, in addition to the DSM-oriented scales depressive, anxious, ADHD, and antisocial. The largest effect sizes were observed in attention problems (*t*(30) = − 5.53, *d* = 1.96, *p*_*FDR*_ = 0.00), externalizing (*t*(30) = − 5.68, *d* = 2.01, *p*_*FDR*_ = 0.00), and antisocial (*t*(30) = − 5.29, *d* = 1.87, *p*_*FDR*_ 0.00). Full results of group comparisons can be found in Table 2. Full results of comparisons between dependent and non-dependent groups and current and previous use can be found in the supplementary material (Table S1 and S2).

In exploratory analyses with categorized T-scores for each ASEBA scale, the AAS group demonstrated a significantly greater proportion of group members with scores over the borderline or clinical thresholds for attention problems (*p*_*FDR*_ = 0.02), internalizing (*p*_*FDR*_ = 0.03), depressive (*p*_*FDR*_ = 0.03), ADHD (*p*_*FDR*_ = 0.02), and antisocial (*p*_*FDR*_ = 0.03) (Table S3, Figure S1). Full results of comparisons between participants with and without dependence are available in the supplementary materials (Table S4).

### AAS dependence

**Fig. 1:**
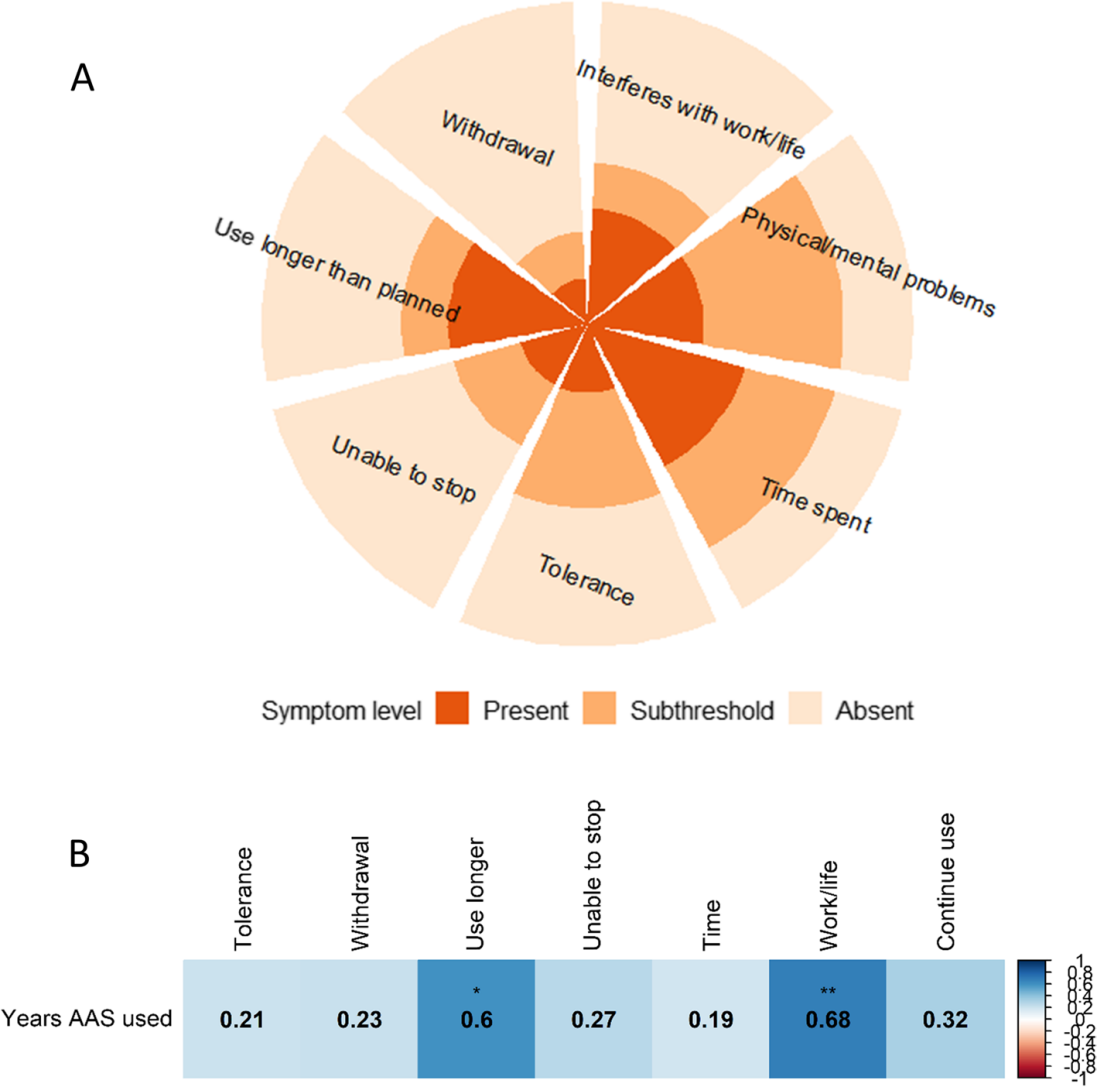
**A** proportion of dependence symptoms reported as absent, subthreshold, or present among females using AAS who completed the clinical interview (*n* = 14). **B** Spearman’s rank correlation of AAS dependence symptoms and total years AAS used (***p < 0.001, **p < 0.01, *p < 0.05)

Based on the diagnostic criteria, seven of the participants were identified as having lifetime AAS dependence (*n* = 7 non-dependent, *n* = 2 missing SCID interview). The most commonly reported dependence symptom was *time spent* (*n* = 7, 50%), and the least frequently reported symptom was *withdrawal* (*n* = 2, 14.3%) (Table 3, Fig. 1A). Positive correlations were identified between total years used and *using longer than planned* (ρ = 0.60, *p* = 0.02) and *interference with work/life activities* (*ρ* = 0.68, *p* = 0.01) (Fig. 1B)*.*

### Buss-Perry, ASEBA, and AAS dependence correlations

**Fig. 2:**
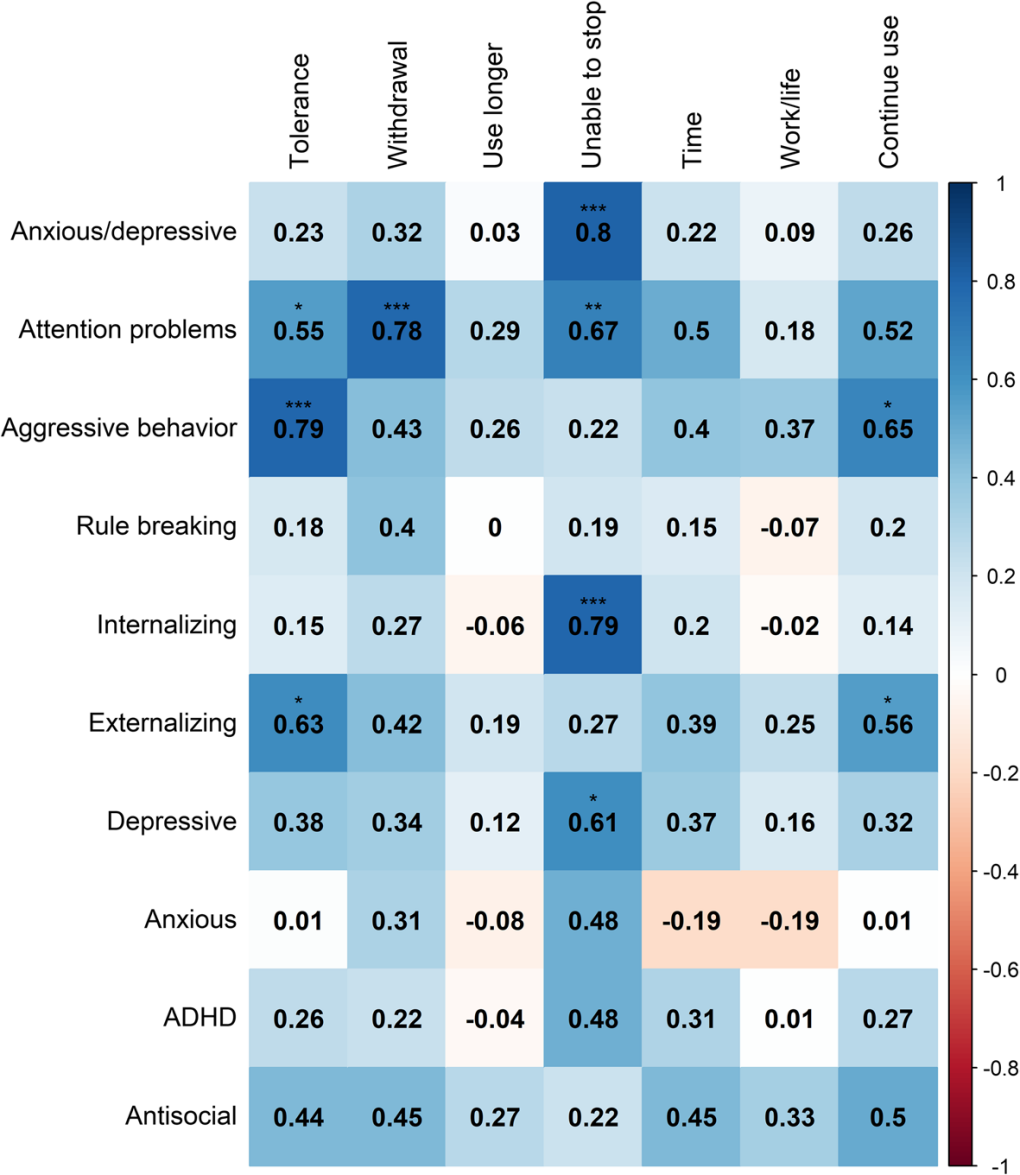
Spearman’s correlation coefficients of ASEBA scales and AAS dependence symptoms. ***p < 0.001, **p < 0.01, *p < 0.05

**Fig. 3:**
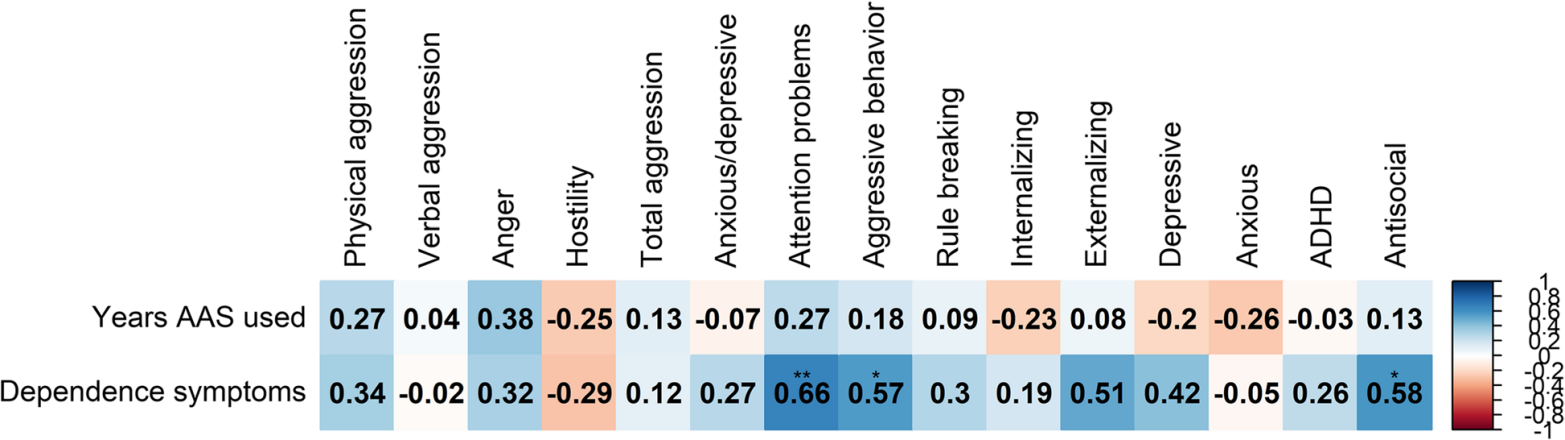
Spearman’s correlation coefficients of ASEBA scales and duration of AAS use and total number of AAS dependence symptoms reported. ***p < 0.001, **p < 0.01, *p < 0.05

In exploratory analyses among participants who had used AAS and completed the SCID-II, significant Spearman’s correlations were identified. The attention problems scale was positively correlated with withdrawal (*ρ* = 0.78, *p* < 0.001), tolerance (*ρ* = 0.55, *p* = 0.04), and being unable to stop use (*ρ* = 0.67, *p* = 0.01). Aggressive behavior was positively correlated with tolerance (*ρ* = 0.79, *p* < 0.001) and continuing to use despite adverse effects (*ρ* = 0.65, *p* = 0.01). Inability to quit use was also correlated with the internalizing (ρ = 0.79, *p* < 0.001), depressive (*ρ* = 0.61, *p* = 0.02) and anxious/depressive scales (*ρ* = 0.80, *p* < 0.001) (Fig. 2).The total number of dependence symptoms experienced were significantly correlated with attention problems (*ρ* = 0.66, *p* = 0.01), aggressive behavior (*ρ* = 0.57, *p* = 0.03), and antisocial (*ρ* = 0.58, *p* = 0.03) (Fig. 3).

## Discussion

The present study found that the most common symptoms of AAS dependence in a sample of weight-lifting women were time spent, and using more/for longer than planned. In addition, women who currently or previously used AAS reported higher levels of aggression, and more internalizing and externalizing problems compared to women who never used AAS. Aggression scales were associated with externalizing behaviors, attention problems and ADHD. Finally, dependence symptoms were associated with anxious/depressive, attention problems, aggressive behavior, internalizing and externalizing scales.

Our findings suggest that the most frequently experienced AAS dependence symptoms differ between males and females who use AAS. Half of the women in our sample reported spending a significant amount of time on activities surrounding AAS use, while only two (14.3%) reported having experienced withdrawal symptoms. By comparison, we previously published findings among male AAS using weightlifters where a similar proportion (41.7%) reported spending significant time on AAS, but a much larger proportion (50.4%) reported withdrawal symptoms [47]. However, it is important to note that time spent includes many aspects of the lifestyle surrounding AAS use, including time training and managing diet and supplement use [29]. We have previously demonstrated that time spent may not be a central symptom in a network of AAS dependence symptoms [47]. Sex differences in AAS dependence symptoms likely reflect sex-based differences in the experience of using AAS, where females may not experience as serious withdrawal due to lower endogenous testosterone. In addition, potential sex differences in time spent may be a result of the relative paucity of information available regarding female AAS use, and increased incidence of treatment seeking behavior among women as a result of AAS-induced side effects [8, 48].

We identified significant differences between women with AAS use and WLC across a number of aggression scales, with a particularly large effect on the anger scale. Our sample indicated particularly high scores compared to previously reported findings of the BPAQ among women using AAS [39]. Surprisingly, the average total aggression score identified among women who reported any AAS use in this study was comparable with the scores reported by males who reported any AAS use [15]). Notably, women demonstrated particularly elevated scores on the anger and hostility subscales. As AAS use is relatively uncommon among females, women who do use these substances may represent an atypical subpopulation with a greater burden of psychological distress relative to men who use AAS, as has been previously suggested [18, 25, 40]. Our findings are consistent with previous research which has indicated increased aggression and labile mood among women who use AAS [37, 38]. Additionally, in a qualitative study women described feeling “grumpier” and finding that they lacked patience while on cycle, in addition to increased anxiety [49].

Externalizing behavior, attention problems and ADHD were among the ASEBA scales with the largest differences between AAS and WLC groups. These findings are in line with previous findings of elevated ADHD symptoms and history of conduct disorder among men using AAS [50, 51]. In addition, ADHD is a well-established risk factor for SUDs [35, 52]. In addition, women who use AAS often use other substances, and exhibit other health-compromising behaviors, which may partially explain the relationship between AAS use and attention problems in this study [44, 53, 54].

While we identified several group differences in maladaptive functioning scales in the current study, it should be noted that there were no statistically significant differences in the adaptive functioning scales. This is somewhat surprising as we would expect that scales on which the AAS group scored particularly high, including ADHD and antisocial, are often associated with increased interpersonal problems [55, 56]. This suggests that this population is somewhat atypical in that they are elevated in maladaptive behavior scales, but comparable to the control group in adaptive functioning. The women in this sample who use AAS appear to have good social support with friends and family, and those that are working or are currently students function well in these roles. It is also likely that a strict training regime and the weight-lifting/bodybuilding community may constitute a protective factor in these areas.

This study cannot determine causality between AAS use and maladaptive behaviors or aggression. While animal studies suggest administering AAS may increase aggression in females [41], evidence from human studies suggest manipulating testosterone does not increase aggression in women [57]. Furthermore, in a sample of males and females using AAS, no significant associations between dose or duration of use and aggression were found [39]. However, it is important to note that aggressive behavior is a complicated phenomenon, which is influenced by interactions between many factors including neurobiological, environmental, and endocrine variables [58]. This complexity contributes to the comorbidities between disorders associated with aggressive behaviors and other psychiatric conditions, which likely explains some of our findings. In addition, SUD is often comorbid with other psychiatric disorders [59], including those related to aggressive behaviors, which may reflect shared underlying mechanisms or vulnerabilities. For example, SUD is more common among people with antisocial personality disorders and oppositional defiant disorder [60, 61]. Previous findings also indicate that females with ADHD are more likely to exhibit aggressive behaviors than females without ADHD [62]. In addition, comorbid ADHD and borderline personality disorder has been associated with high levels of impulsivity and aggression compared to participants with either disorder alone [63]. In a previous study of the same sample we identified higher levels of borderline personality disorder symptoms among women who used AAS compared to WLC, thus it is feasible that this comorbidity exists within our study sample and may contribute to the elevated aggression scales observed.

While exploratory, the correlation analyses suggest that attention problems and aggressive behavior are correlated both with specific symptoms of AAS dependence, including tolerance, and with the total number of dependence symptoms reported. Previous studies indicate associations between increased drug dependence duration and complexity (number of SUDs) among SUD patients with ADHD, and it is possible that this is the case for AAS dependence as well [64]. In addition, tolerance was correlated with attention problems, aggressive behavior, and externalizing behaviors, which may suggest a dose–response as dose is increased as a result of developing tolerance. Being unable to stop AAS use was associated with internalizing scales, which suggests that attempting to quit may increase depression or anxiety symptoms, or that being unable to quit contributes to these internalizing symptoms.

## Limitations

A notable limitation of this study is the small sample size. However, there is a lack of research regarding AAS use and particularly dependence among females, and we believe it is important to report these exploratory findings in an effort to increase understanding of the potential risk factors and consequences of AAS use for females seeking information. In addition, the variation in time scale of the metrics included (i.e. lifetime dependence, ASEBA based on previous six months) indicates that the results presented should be interpreted with caution. Current and previous use were taken into account in additional analyses, as some participants may be classified as “dependent” while not currently using, and vice versa, which may have an impact on the findings. In addition, the AAS using group comprises women with a wide range of years of cumulative use (1–26 years). The study also relies on self-report measures, including the ASEBA and BPAQ, with some scales of the ASEBA having less than acceptable reliability as indicated by Cronbach’s alpha. The categorization of the ASEBA scalers indicate normal/borderline/clinical ranges, however the women in this study were not formally evaluated or diagnosed with ADHD or other disorders. Finally, due to the cross-sectional nature of this study, the direction of the relationships cannot be determined.

## Conclusion

Based on our findings, attention problems and ADHD symptoms are likely associated with both AAS use and dependence in females, as has been observed among male users. While the current study cannot establish causality, it is likely that some symptoms of behavioral and psychological problems precede AAS use, and are therefore relevant targets for prevention efforts. Recognizing and treating symptoms of psychiatric disorders may help with cessation of AAS use and alleviating symptoms of dependence. Furthermore, AAS dependence likely differs between men and women, which may reflect differences in biological response to exogenous androgens. Sex differences should be taken into account when evaluating AAS dependence, and consideration should be given to the relevance of certain symptoms when determining clinical criteria for males or females who use AAS, as withdrawal likely plays a less central role for women. Similarly, while aggressive behaviors are more often associated with men, our findings indicate similar levels of aggressive traits based on self-report questionnaires between men and women, which clinicians should be aware of. Clinicians should also be aware of the complex challenges women who use AAS face, including both their own psychological distress as well as the additional stigma experienced, particularly as women who use AAS are more likely to seek health services than men.

## Supplementary Information

Supplementary Material 1.

## Data Availability

The datasets generated and/or analysed during the current study are not publicly available due to the sensitive nature of the data but are available from the corresponding author on reasonable request.
