# Supplementary material for: Psychological traits associated with anabolic androgenic steroid use and dependence: an exploratory cross-sectional study among female athletes
Source: BMC Womens Health. 2025 May 5;25:214. doi: 10.1186/s12905-025-03711-5 (PMC12051273; doi:10.1186/s12905-025-03711-5)
Supplement: Supplementary file 1 — Supplementary Material 1. [file 12905_2025_3711_MOESM1_ESM.docx]

**Supplementary materials**

**Table S1**. Statistical comparisons between AAS-dependent and non-dependent participants

|  | **Mean (SD)** | **Mean (SD)** | **t** | **d** | ***p*** | ***p*_FDR_** |
| --- | --- | --- | --- | --- | --- | --- |
|  | **Non-dependent, n=7** | **Dependent, N=7** |  |  |  |  |
| **Buss-Perry^a^** |  |  |  |  |  |  |
| Physical aggression | 20.50 (5.79) | 26.83 (11.51) | -1.20 | -0.70 | 0.27 | 0.49 |
| Verbal aggression | 17.67 (4.93) | 19.67 (6.59) | -0.60 | -0.34 | 0.57 | 0.77 |
| Anger | 24.83 (7.52) | 27.50 (9.16) | -0.55 | -0.32 | 0.59 | 0.77 |
| Hostility | 27.33 (11.84) | 21.17 (9.15) | 1.01 | 0.58 | 0.34 | 0.55 |
| Total aggression | 90.33 (22.93) | 95.17 (16.36) | -0.42 | -0.24 | 0.68 | 0.85 |
| **ASEBA (T-scores)** |  |  |  |  |  |  |
| Anxious/depressive | 59.57 (6.29) | 69.86 (16.81) | -1.52 | -0.81 | 0.17 | 0.49 |
| Withdrawn | 59.57 (11.22) | 58.57 (8.73) | 0.19 | 0.10 | 0.86 | 0.86 |
| Somatic complaints | 55.29 (6.87) | 64.71 (13.34) | -1.66 | -0.89 | 0.13 | 0.49 |
| Thought problems | 55.00 (6.51) | 59.71 (7.34) | -1.27 | -0.68 | 0.23 | 0.49 |
| Attention problems | 61.14 (6.52) | 68.29 (6.18) | -2.10 | -1.12 | 0.06 | 0.49 |
| Aggressive behavior | 56.86 (7.08) | 65.43 (7.91) | -2.14 | -1.14 | **0.05** | 0.49 |
| Rule breaking | 58.71 (5.47) | 64.00 (11.20) | -1.12 | -0.60 | 0.29 | 0.51 |
| Intrusive | 52.29 (4.75) | 57.14 (5.24) | -1.82 | -0.97 | 0.09 | 0.49 |
| Internalizing | 56.57 (11.96) | 66.71 (16.11) | -1.34 | -0.71 | 0.21 | 0.49 |
| Externalizing | 55.43 (8.24) | 66.14 (9.96) | -2.19 | -1.17 | **0.05** | 0.49 |
| Depressive | 58.00 (8.19) | 69.00 (12.96) | -1.90 | -1.01 | 0.09 | 0.49 |
| Anxious | 54.43 (5.29) | 58.86 (12.27) | -0.877 | -0.469 | 0.41 | 0.62 |
| Somatic problems | 53.29 (5.31) | 60.29 (14.04) | -1.23 | -0.659 | 0.25 | 0.49 |
| Avoidant | 58.86 (5.90) | 57.57 (9.05) | 0.315 | 0.168 | 0.76 | 0.86 |
| ADHD | 65.14 (12.28) | 72.57 (8.10) | -1.34 | -0.714 | 0.21 | 0.49 |
| Antisocial | 59.00 (6.71) | 66.57 (10.05) | -1.66 | -0.886 | 0.13 | 0.49 |
| Friends | 49.14 (7.76) | 50.14 (8.75) | -0.23 | -0.12 | 0.82 | 0.86 |
| Spouse/partner^b^ | 54.00 (6.93) | 46.00 (5.66) | 1.58 | 1.20 | 0.24 | 0.49 |
| Family | 49.29 (8.58) | 45.29 (9.72) | 0.82 | 0.44 | 0.43 | 0.62 |
| Job^c^ | 45.83 (5.46) | 47.33 (8.08) | -0.29 | -0.24 | 0.79 | 0.86 |
| Education^d^ | 47.50 (3.54) | 49.00 (7.55) | -0.30 | -0.23 | 0.79 | 0.86 |
|  |  |  |  |  |  |  |
| t= Welch’s t-statistic, p adjusted= false discovery rate adjusted p-value, T= T-score, a= Missing n=1 non-dependent, n= 1 dependent, b = no spouse/partner n = 2 non-dependent, n = 5 dependent, c = not currently working n = 1 non-dependent, n = 4 dependent, d = not currently a student n = 5 non-dependent, n = 4 dependent | | | | | | |

**Table S2**. Statistical comparisons between participants who currently and previously used AAS

|  | **Mean (SD)** | **Mean (SD)** | **t** | **d** | ***p*** | ***p*_FDR_** |
| --- | --- | --- | --- | --- | --- | --- |
|  | **Previous, n=9** | **Current, N=7** |  |  |  |  |
| **Buss-Perry^a^** |  |  |  |  |  |  |
| Physical aggression | 23.88 (9.52) | 23.17 (8.54) | 0.15 | 0.08 | 0.89 | 0.92 |
| Verbal aggression | 19.00 (4.87) | 18.50 (6.09) | 0.17 | 0.09 | 0.87 | 0.92 |
| Anger | 23.12 (7.86) | 27.50 (8.26) | -1.00 | -0.54 | 0.34 | 0.92 |
| Hostility | 23.50 (6.65) | 25.33 (13.60) | -0.30 | -0.18 | 0.77 | 0.92 |
| Total aggression | 89.50 (13.80) | 94.50 (24.01) | -0.46 | -0.27 | 0.66 | 0.92 |
| **ASEBA (T-scores)** |  |  |  |  |  |  |
| Anxious/depressive | 66.78 (13.88) | 63.29 (11.16) | 0.56 | 0.27 | 0.59 | 0.92 |
| Withdrawn | 57.33 (6.86) | 60.29 (11.79) | -0.59 | -0.32 | 0.57 | 0.92 |
| Somatic complaints | 63.00 (11.97) | 58.14 (9.65) | 0.90 | 0.44 | 0.38 | 0.92 |
| Thought problems | 59.11 (7.42) | 55.43 (6.43) | 1.06 | 0.53 | 0.31 | 0.92 |
| Attention problems | 66.11 (5.28) | 62.71 (8.30) | 0.94 | 0.50 | 0.37 | 0.92 |
| Aggressive behavior | 60.89 (8.58) | 60.00 (8.23) | 0.21 | 0.11 | 0.84 | 0.92 |
| Rule breaking | 60.00 (10.19) | 60.86 (7.40) | -0.19 | -0.09 | 0.85 | 0.92 |
| Intrusive | 55.33 (5.48) | 53.00 (4.90) | 0.90 | 0.45 | 0.39 | 0.92 |
| Internalizing | 64.22 (13.15) | 59.86 (15.10) | 0.61 | 0.31 | 0.56 | 0.92 |
| Externalizing | 60.44 (11.16) | 59.00 (9.63) | 0.28 | 0.14 | 0.79 | 0.92 |
| Depressive | 66.11 (12.72) | 62.57 (10.13) | 0.62 | 0.30 | 0.55 | 0.92 |
| Anxious | 59.11 (9.68) | 55.71 (8.69) | 0.74 | 0.37 | 0.47 | 0.92 |
| Somatic problems | 59.00 (11.62) | 55.14 (9.03) | 0.75 | 0.36 | 0.47 | 0.92 |
| Avoidant | 57.00 (8.41) | 57.71 (6.18) | -0.20 | -0.09 | 0.85 | 0.92 |
| ADHD | 68.44 (7.20) | 68.43 (13.67) | 0.00 | 0.00 | 1.00 | 0.92 |
| Antisocial | 63.33 (9.91) | 61.71 (6.87) | 0.39 | 0.19 | 0.71 | 0.92 |
| Friends | 50.89 (8.37) | 49.29 (7.72) | 0.40 | 0.20 | 0.70 | 0.92 |
| Spouse/partner^b^ | 48.00 (8.16) | 54.00 (4.90) | -1.26 | -0.89 | 0.26 | 0.92 |
| Family | 43.00 (11.87) | 48.00 (9.59) | -0.93 | -0.46 | 0.37 | 0.92 |
| Job^c^ | 44.20 (6.87) | 46.67 (6.02) | -0.63 | -0.38 | 0.55 | 0.92 |
| Education^d^ | 45.75 (8.96) | 47.50 (3.54) | -0.34 | -0.22 | 0.75 | 0.92 |
|  |  |  |  |  |  |  |
| t= Welch’s t-statistic, p adjusted= false discovery rate adjusted p-value, T= T-score, a= Missing n=1 previous, n= 1 current, b = no spouse/partner n = 5 previous, n = 3 current, c = not currently working n = 4 previous, n = 1 current, d = not currently a student n = 5 previous, n = 5 current | | | | | | |

**Table S3**. Proportion of participants in normal, borderline, and clinical ranges of ASEBA scales among AAS and WLC groups

|  | Level | WLC | AAS | p | p_FDR_ |
| --- | --- | --- | --- | --- | --- |
| n |  | 16 | 16 |  |  |
| Anxious/depressive (%) | Normal | 15 ( 93.8) | 9 ( 56.2) | 0.065 | 0.171 |
|  | Borderline | 1 ( 6.2) | 4 ( 25.0) |  |  |
|  | Clinical | 0 ( 0.0) | 3 ( 18.8) |  |  |
| Withdrawn (%) | Normal | 15 ( 93.8) | 12 ( 75.0) | 0.226 | 0.339 |
|  | Borderline | 1 ( 6.2) | 1 ( 6.2) |  |  |
|  | Clinical | 0 ( 0.0) | 3 ( 18.8) |  |  |
| Somatic complaints (%) | Normal | 15 ( 93.8) | 12 ( 75.0) | 0.226 | 0.339 |
|  | Borderline | 1 ( 6.2) | 1 ( 6.2) |  |  |
|  | Clinical | 0 ( 0.0) | 3 ( 18.8) |  |  |
| Thought problems (%) | Normal | 15 ( 93.8) | 11 ( 68.8) | 0.172 | 0.339 |
|  | Borderline | 1 ( 6.2) | 4 ( 25.0) |  |  |
|  | Clinical | 0 ( 0.0) | 1 ( 6.2) |  |  |
| Attention problems (%) | Normal | 16 (100.0) | 7 ( 43.8) | 0.001 | 0.021 |
|  | Borderline | 0 ( 0.0) | 5 ( 31.2) |  |  |
|  | Clinical | 0 ( 0.0) | 4 ( 25.0) |  |  |
| Aggressive behavior (%) | Normal | 16 (100.0) | 10 ( 62.5) | 0.018 | 0.063 |
|  | Borderline | 0 ( 0.0) | 3 ( 18.8) |  |  |
|  | Clinical | 0 ( 0.0) | 3 ( 18.8) |  |  |
| Rule breaking (%) | Normal | 16 (100.0) | 12 ( 75.0) | 0.101 | 0.236 |
|  | Borderline | 0 ( 0.0) | 1 ( 6.2) |  |  |
|  | Clinical | 0 ( 0.0) | 3 ( 18.8) |  |  |
| Intrusive (%) | Normal | 16 (100.0) | 15 ( 93.8) | 1 | 1 |
|  | Borderline | 0 ( 0.0) | 1 ( 6.2) |  |  |
|  | Clinical | 0 ( 0.0) | 0 ( 0.0) |  |  |
| Internalizing (%) | Normal | 16 (100.0) | 9 ( 56.2) | 0.007 | 0.029 |
|  | Borderline | 0 ( 0.0) | 4 ( 25.0) |  |  |
|  | Clinical | 0 ( 0.0) | 3 ( 18.8) |  |  |
| Externalizing (%) | Normal | 16 (100.0) | 11 ( 68.8) | 0.043 | 0.129 |
|  | Borderline | 0 ( 0.0) | 3 ( 18.8) |  |  |
|  | Clinical | 0 ( 0.0) | 2 ( 12.5) |  |  |
| Friends (%) | Normal | 16 (100.0) | 16 (100.0) | 1 | 1 |
|  | Borderline | 0 ( 0.0) | 0 ( 0.0) |  |  |
|  | Clinical | 0 ( 0.0) | 0 ( 0.0) |  |  |
| Spouse/partner (%) | Normal | 10 (100.0) | 8 (100.0) | 1 | 1 |
|  | Borderline | 0 ( 0.0) | 0 ( 0.0) |  |  |
|  | Clinical | 0 ( 0.0) | 0 ( 0.0) |  |  |
| Family (%) | Normal | 14 ( 87.5) | 14 ( 87.5) | 1 | 1 |
|  | Borderline | 0 ( 0.0) | 0 ( 0.0) |  |  |
|  | Clinical | 2 ( 12.5) | 2 ( 12.5) |  |  |
| Job (%) | Normal | 15 ( 93.8) | 11 (100.0) | 1 | 1 |
|  | Borderline | 1 ( 6.2) | 0 ( 0.0) |  |  |
|  | Clinical | 0 ( 0.0) | 0 ( 0.0) |  |  |
| Education (%) | Normal | 5 ( 83.3) | 6 (100.0) | 1 | 1 |
|  | Borderline | 0 ( 0.0) | 0 ( 0.0) |  |  |
|  | Clinical | 1 ( 16.7) | 0 ( 0.0) |  |  |
| Depressive (%) | Normal | 15 ( 93.8) | 7 ( 43.8) | 0.005 | 0.029 |
|  | Borderline | 1 ( 6.2) | 3 ( 18.8) |  |  |
|  | Clinical | 0 ( 0.0) | 6 ( 37.5) |  |  |
| Anxious (%) | Normal | 16 (100.0) | 13 ( 81.2) | 0.226 | 0.339 |
|  | Borderline | 0 ( 0.0) | 1 ( 6.2) |  |  |
|  | Clinical | 0 ( 0.0) | 2 ( 12.5) |  |  |
| Somatic problems (%) | Normal | 15 ( 93.8) | 13 ( 81.2) | 0.733 | 1 |
|  | Borderline | 1 ( 6.2) | 1 ( 6.2) |  |  |
|  | Clinical | 0 ( 0.0) | 2 ( 12.5) |  |  |
| Avoidant (%) | Normal | 16 (100.0) | 13 ( 81.2) | 0.226 | 0.339 |
|  | Borderline | 0 ( 0.0) | 2 ( 12.5) |  |  |
|  | Clinical | 0 ( 0.0) | 1 ( 6.2) |  |  |
| ADHD (%) | Normal | 14 ( 87.5) | 7 ( 43.8) | 0.002 | 0.021 |
|  | Borderline | 2 ( 12.5) | 1 ( 6.2) |  |  |
|  | Clinical | 0 ( 0.0) | 8 ( 50.0) |  |  |
| Antisocial (%) | Normal | 16 (100.0) | 9 ( 56.2) | 0.007 | 0.029 |
|  | Borderline | 0 ( 0.0) | 5 ( 31.2) |  |  |
|  | Clinical | 0 ( 0.0) | 2 ( 12.5) |  |  |

**Table S4**. Proportion of participants in normal, borderline, and clinical ranges of ASEBA scales among AAS-dependent and non-dependent participants

|  | level | Non-dependent | Dependent | p | p_FDR_ |
| --- | --- | --- | --- | --- | --- |
| n |  | 7 | 7 |  |  |
| Anxious/depressive (%) | Normal | 5 ( 71.4) | 4 ( 57.1) | 0.559 | 0.979 |
|  | Borderline | 2 ( 28.6) | 1 ( 14.3) |  |  |
|  | Clinical | 0 ( 0.0) | 2 ( 28.6) |  |  |
| Withdrawn (%) | Normal | 5 ( 71.4) | 5 ( 71.4) | 1 | 1 |
|  | Borderline | 0 ( 0.0) | 1 ( 14.3) |  |  |
|  | Clinical | 2 ( 28.6) | 1 ( 14.3) |  |  |
| Somatic complaints (%) | Normal | 6 ( 85.7) | 5 ( 71.4) | 0.462 | 0.979 |
|  | Borderline | 1 ( 14.3) | 0 ( 0.0) |  |  |
|  | Clinical | 0 ( 0.0) | 2 ( 28.6) |  |  |
| Thought problems (%) | Normal | 6 ( 85.7) | 4 ( 57.1) | 0.559 | 0.979 |
|  | Borderline | 1 ( 14.3) | 2 ( 28.6) |  |  |
|  | Clinical | 0 ( 0.0) | 1 ( 14.3) |  |  |
| Attention problems (%) | Normal | 4 ( 57.1) | 2 ( 28.6) | 0.128 | 0.979 |
|  | Borderline | 3 ( 42.9) | 1 ( 14.3) |  |  |
|  | Clinical | 0 ( 0.0) | 4 ( 57.1) |  |  |
| Aggressive behavior (%) | Normal | 5 ( 71.4) | 3 ( 42.9) | 0.339 | 0.979 |
|  | Borderline | 2 ( 28.6) | 1 ( 14.3) |  |  |
|  | Clinical | 0 ( 0.0) | 3 ( 42.9) |  |  |
| Rule breaking (%) | Normal | 6 ( 85.7) | 4 ( 57.1) | 0.559 | 0.979 |
|  | Borderline | 0 ( 0.0) | 1 ( 14.3) |  |  |
|  | Clinical | 1 ( 14.3) | 2 ( 28.6) |  |  |
| Intrusive (%) | Normal | 7 (100.0) | 6 ( 85.7) | 1 | 1 |
|  | Borderline | 0 ( 0.0) | 1 ( 14.3) |  |  |
|  | Clinical | 0 ( 0.0) | 0 ( 0.0) |  |  |
| Internalizing (%) | Normal | 5 ( 71.4) | 4 ( 57.1) | 1 | 1 |
|  | Borderline | 1 ( 14.3) | 1 ( 14.3) |  |  |
|  | Clinical | 1 ( 14.3) | 2 ( 28.6) |  |  |
| Externalizing (%) | Normal | 6 ( 85.7) | 3 ( 42.9) | 0.339 | 0.979 |
|  | Borderline | 1 ( 14.3) | 2 ( 28.6) |  |  |
|  | Clinical | 0 ( 0.0) | 2 ( 28.6) |  |  |
| Friends (%) | Normal | 7 (100.0) | 7 (100.0) | 1 | 1 |
|  | Borderline | 0 ( 0.0) | 0 ( 0.0) |  |  |
|  | Clinical | 0 ( 0.0) | 0 ( 0.0) |  |  |
| Spouse/partner (%) | Normal | 5 (100.0) | 2 (100.0) | 1 | 1 |
|  | Borderline | 0 ( 0.0) | 0 ( 0.0) |  |  |
|  | Clinical | 0 ( 0.0) | 0 ( 0.0) |  |  |
| Family (%) | Normal | 7 (100.0) | 6 ( 85.7) | 1 | 1 |
|  | Borderline | 0 ( 0.0) | 0 ( 0.0) |  |  |
|  | Clinical | 0 ( 0.0) | 1 ( 14.3) |  |  |
| Job (%) | Normal | 6 (100.0) | 3 (100.0) | 1 | 1 |
|  | Borderline | 0 ( 0.0) | 0 ( 0.0) |  |  |
|  | Clinical | 0 ( 0.0) | 0 ( 0.0) |  |  |
| Education (%) | Normal | 2 (100.0) | 3 (100.0) | 1 | 1 |
|  | Borderline | 0 ( 0.0) | 0 ( 0.0) |  |  |
|  | Clinical | 0 ( 0.0) | 0 ( 0.0) |  |  |
| Depressive (%) | Normal | 5 ( 71.4) | 2 ( 28.6) | 0.388 | 0.979 |
|  | Borderline | 1 ( 14.3) | 2 ( 28.6) |  |  |
|  | Clinical | 1 ( 14.3) | 3 ( 42.9) |  |  |
| Anxious (%) | Normal | 7 (100.0) | 5 ( 71.4) | 0.462 | 0.979 |
|  | Borderline | 0 ( 0.0) | 0 ( 0.0) |  |  |
|  | Clinical | 0 ( 0.0) | 2 ( 28.6) |  |  |
| Somatic problems (%) | Normal | 7 (100.0) | 5 ( 71.4) | 0.462 | 0.979 |
|  | Borderline | 0 ( 0.0) | 0 ( 0.0) |  |  |
|  | Clinical | 0 ( 0.0) | 2 ( 28.6) |  |  |
| Avoidant (%) | Normal | 6 ( 85.7) | 5 ( 71.4) | 1 | 1 |
|  | Borderline | 1 ( 14.3) | 1 ( 14.3) |  |  |
|  | Clinical | 0 ( 0.0) | 1 ( 14.3) |  |  |
| ADHD (%) | Normal | 4 ( 57.1) | 2 ( 28.6) | 0.592 | 0.979 |
|  | Borderline | 0 ( 0.0) | 0 ( 0.0) |  |  |
|  | Clinical | 3 ( 42.9) | 5 ( 71.4) |  |  |
| Antisocial (%) | Normal | 5 ( 71.4) | 2 ( 28.6) | 0.388 | 0.979 |
|  | Borderline | 2 ( 28.6) | 3 ( 42.9) |  |  |
|  | Clinical | 0 ( 0.0) | 2 ( 28.6) |  |  |

**Figure S1**. Left side: proportion of AAS and WLC with normal, borderline, and clinical levels of ASEBA scales. Right side: proportion of AAS dependent and non-dependent groups with normal, borderline, and clinical levels of ASEBA scales.
